# Aberrant expression of SPAG6 and NM23 predicts poor prognosis of human osteosarcoma

**DOI:** 10.3389/fgene.2022.1012548

**Published:** 2022-09-19

**Authors:** Zhengqi Bao, Ruizhi Zhu, Huagang Fan, Yuchen Ye, Tian Li, Damin Chai

**Affiliations:** ^1^ Department of Orthopedics, The First Affiliated Hospital of Bengbu Medical University, Bengbu, China; ^2^ Anhui Province Key Laboratory of Tissue Transplantation, Bengbu Medical University, Bengbu, China; ^3^ School of Basic Medicine, Fourth Military Medical University, Xi’an, China; ^4^ Department of Pathology, The First Affiliated Hospital of Bengbu Medical University, Bengbu, China

**Keywords:** osteosarcoma, SPAG6, NM23, prognosis, overall survival

## Abstract

**Objective:** To investigate the expression and clinical significance of sperm-associated antigen 6 and NM23 proteins in human osteosarcoma.

**Methods:** The specimens of conventional osteosarcoma with follow-up from 42 Chinese patients were analyzed in this study, and 12 cases of osteochondroma were considered controls. The expression of SPAG6 and NM23 was inspected using immunohistochemical staining, qRT-PCR, and Western blotting methods.

**Results:** The positive expression rate of SPAG6 protein (71.43%) in 42 cases of osteosarcoma tissue was significantly higher than that (33.33%) in 12 cases of osteochondroma tissues (*p* < 0.05), while the positive rate of NM23 protein (35.71%) in osteosarcoma tissue was lower than that (58.33%) in osteochondroma tissue (*p* < 0.05). The mRNA and protein levels of SPAG6 were significantly higher than those of the adjacent normal tissues, while the expression of NM23 was lower in osteosarcoma tissues than that in the controls (*p* < 0.05 for all). There was a positive relationship between the expression of SPAG6 and pathological grade, metastasis, and Enneking stage (*p* < 0.05 for all). The overall survival rate of osteosarcoma patients with SPAG6 positive expression was significantly lower than that with SPAG6 negative expression. The relationship between the expression of NM23 and pathological grade, metastasis, and Enneking stage was negative (*p* < 0.05 for all). The overall survival rate of the osteosarcoma patients with NM23 positive expression was higher than that of the patients with NM23 negative expression (*p* < 0.05).

**Conclusion:** Overexpression of SPAG6 and low expression of NM23 are negatively related to pathological grade, metastasis, and Enneking stage and prognosis of osteosarcoma patients. This suggested that SPAG6 and NM23 should be considered candidate prognostic biomarkers for patients with osteosarcoma.

## Introduction

Osteosarcoma (OS), also referred to as osteogenic sarcoma, is the most popular malignant tumor originating in the bones and is more prevalent in males than in females (Mirabello et al., 2009a; Mirabello et al., 2009b; Biermann et al., 2013). The worldwide incidence of osteosarcoma is about 3–4 cases per million each year, about 800 new cases are diagnosed, and half of these are reported in adolescents (Rickel et al., 2017; Assi et al., 2021). Patients with osteosarcoma often have high cancer-related mortality, especially once metastasis to the lungs begins, the 5-year survival rate becomes less than 20% (EESNW Group, 2012; Luetke et al., 2014). In addition to surgery, the use of neoadjuvant chemotherapy and immunotherapy significantly increases the 5-year survival of non-metastasis osteosarcoma patients to approximately 58–75% (Bacci et al., 2005). However, there have been only minimal improvements in the prognosis of metastasis osteosarcoma patients in the last 2 decades (EESNW Group, 2012). Therefore, it is necessary to research novel molecular markers of metastasis and to identify the underlying molecular mechanism of OS evolvement and progression, which will identify a novel therapeutic strategy for patients with OS.

Sperm-associated antigen 6 (SPAG6) gene, first identified in human testicular tissue, also named CT141 and pf16, is considered a cancer-testis antigen (CTA) and involved in many cancers (Abe et al., 2008; Silina et al., 2011; Coan et al., 2018; Jiang et al., 2019). Many studies’ results have shown that SPAG6 plays a critical role in tumor involvement and progression, especially in myelodysplastic syndrome (MDSC), the common hematological malignancies, and SPAG6 levels were significantly higher than those in solid tumors (Barretina et al., 2019). The aberrant expression of SPAG6 has been found in neuroblastoma (NBL) cell lines, serous ovarian cancer (HGSOC) samples, myeloid SKM-1 and K562 cell lines, breast cancer tissues, bladder cancer tissues, and non-small-cell lung cancer (NSCLC) samples (Abe et al., 2008; Kitchen et al., 2015; Altenberger et al., 2017; Coan et al., 2018; Jiang et al., 2019). Non-metastasis 23 (NM23) plays a suppressive role in tumor metastasis (Carotenuto et al., 2015). Studies have confirmed that the low expression of NM23 is positively related to metastasis and irradiation (Xi et al., 2019; Wang et al., 2021). Until now, there are few reports about the association of the expression of SPAG6 and NM23 with the metastasis and prognosis of osteosarcoma, and the precise role of SPAG6 is unclear.

In this study, we detected the association between SPAG6 and NM23 and the clinicopathological parameters of metastasis and prognosis of osteosarcoma using immunohistochemical staining, qRT-PCR, and Western blotting methods.

## Materials and methods

### Ethics statement and human osteosarcoma tissue sample collection

Ethical approval for the overall study was agreed upon by the Ethics Committee of The First Affiliated Hospital of Bengbu Medical College, and all the patients consented to the study. A total of 42 cases of paraffin-embedded paired conventional human osteosarcoma tissues were collected for this retrospective review, during the time of surgery from January 2005 to December 2016 at The First Affiliated Hospital of Bengbu Medical College. A total of 12 osteochondroma samples were obtained as controls for the study. Also, from six cases of human osteosarcoma, fresh osteosarcoma specimens and adjacent normal muscle tissues (control) for qRT-PCR and Western blotting (Table 1) were immediately put in liquid nitrogen, frozen, and stored at −80°C. All the human osteosarcoma patients in this study were managed with surgery but did not manage with chemotherapy or radiotherapy. Relevant clinicopathological parameters were obtained by retrospectively reviewing the patients’ medical records. The survival time of the patients with osteosarcoma in this study was predicted by the follow-up through phone or through out-patient visiting or through consulting local police stations, and the time interval was 3 months until the patients died or Jun 2022.

**TABLE 1:** Clinicopathological data of the human conventional osteosarcoma patients with their lesions detected using qRT-PCR and Western blotting (*n* = 6).

Patient code	Gender	Age (years)	Site	Histological type	Metastasis	Diameter (cm)	Pathological degree	Enneking stage
1	Female	6	Femur	Osteoblast	No	3.5	G1	Ⅰ
2	Male	21	Humerus	Osteoblast	Lung	5.4	G2	Ⅲ
3	Male	28	Thurl	Fibroblast	Lung	6.3	G2	Ⅲ
4	Female	36	Femur	Osteoblast	Lung	4.5	G2	Ⅲ
5	Male	48	Jaw	Fibroblast	No	2.5	G1	Ⅰ
6	Male	22	Femur	Mixed	Lung	5.5	G2	Ⅲ

### Immunohistochemical analysis and interpretation

Immunohistochemical staining methods were performed to detect the expression of SPAG6 in specimens of the 42 human osteosarcoma and control tissues as described previously according to the procedure of the Elivision TM Plus detection kit (Lab Vision, United States) (Yang et al., 2014; Bao et al., 2016). Fixed in 10% buffered formalin, all of the tissues were paraffin-embedded and sliced into 3–4-μm-thick tissue sections. The expression of SPAG6 (dilution: 1:200, Catalog No. Ab155653, Abcam, United States) and NM23 (dilution: 1:100, Catalog No. 3338, Cell Signaling Technology, Inc., Danvers, MA, United States) was predominantly localized in the cytoplasm. Upon immunohistochemical staining, the immunoreaction was scored based on the percentage of positively stained tumor cells: 1 (<10%); 2 (11%–50%); 3 (51%–75%); and 4 (>75%). The intensity was scored as follows: 0 (negative); 1 (weak); 1 (moderate); and 1 (strong). The final score was decided by multiplying the staining percentage by the intensity, which commonly ranges from 0 to 12. The score was interpreted as >2, which is a positive expression.

### Real-time PCR

The total RNA of fresh osteosarcoma tissues and cells in the test and control group was obtained using TRIzol (Invitrogen, Carlsbad) and then reverse-transcribed to cDNA using the reverse transcription system (Bao et al., 2019). The expression of SPAG6 was determined using the SYBR Green RT-PCR Assay (Takara, Dalian, China) and normalized to GAPDH (glyceraldehyde 3-phosphate dehydrogenase) as described previously (Ma et al., 2019). Quantitative real-time PCR was implemented on an ABI 7900 System with SYBR Green (Takara, Dalian, China). The primers used in the PCR are as follows: SPAG6, forward primer, 5′-AGC AAT GGC AGT CAT CAT TTC-3′ and reverse primer, 5′-GGA TGA ATG GTC GGG AAC TT- 3′; NM23, forward primer, 5′-ACG​CTT​GCT​CTG​TTT​GTG​G-3′ and reverse primer, 5′-CTG​GAA​GGC​ACA​CCA​TCC-3'; and GAPDH, forward primer, 5-CAG CCT CAA GAT CAGCA-30 and reverse primer, 5′-TGT GGT CAT GAG TCC TTC CA-3′.

### Western blot analyses

Western blot analyses were performed as reported previously (Du et al., 2015; Bao et al., 2016). Briefly, human osteosarcoma and normal adjacent soft tissues were rinsed two times with ice-cold phosphate-buffered saline (PBS, pH 7.4) and lysed with RIPA buffer supplemented with protease inhibitors. Then, the lysates were boiled for 10 min in a water bath and centrifuged at 12,000 g at 4°C for about 10 min. The protein concentrations in the supernatant were surveyed by bicinchoninic acid (BCA) protein assay (Pierce, United States). Equal amounts of proteins were loaded on 10% sodium dodecyl sulfate-polyacrylamide gel electrophoresis (SDS-PAGE) and transferred to nitrocellulose membranes (Hybond ECL, GE Healthcare Bio-Sciences Corp., United States). The membranes were then blocked with 5% non-fat milk, and protein expression was measured as described previously (Chai et al., 2019; Ma et al., 2019).

### Statistical analysis

Statistical analysis was performed using SPSS version 19.0 software (SPSS Inc., United States) and GraphPad Prism version 5 for Windows (GraphPad Software, San Diego, California, United States). All data were presented as mean ± SD. Comparisons between groups were implemented by one-way ANOVA followed by Tukey’s *post hoc* test. The correlation between SPAG6 and NM23 was analyzed using Spearman correlation analysis. The relationship between SPAG6 and overall survival time of patients with human osteosarcoma was performed using the Kaplan−Meier method and log-rank test. Multivariate prognosis analysis was carried out using the Cox regression method. The error bars represent SD. *p* < 0.05 was considered statistically significant.

## Results

### Aberrant expression of SPAG6 and NM23 in osteosarcoma tissue

**FIGURE 1:**
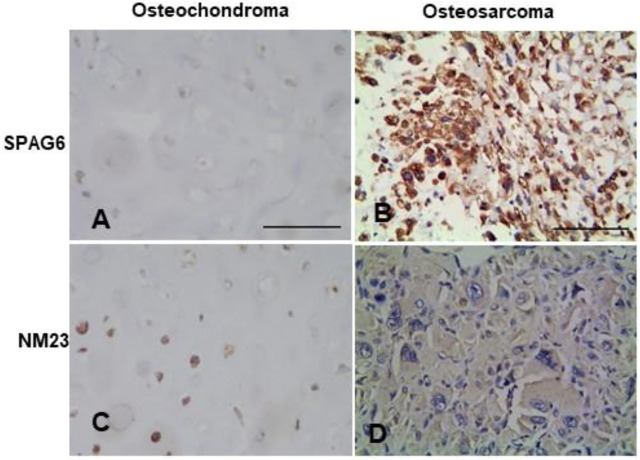
Representation micrographs showing SPAG6 and NM23 proteins in osteochondroma and osteosarcoma tissues (Elivision). **(A,B)** SPAG6 staining is predominantly localized in the cytoplasm in the osteochondroma tissue and osteosarcoma tissue. **(C,D)** NM23 staining is predominantly localized in the cytoplasm in the osteochondroma tissue and osteosarcoma tissue. Scale bar = 50 μm.

**FIGURE 2:**
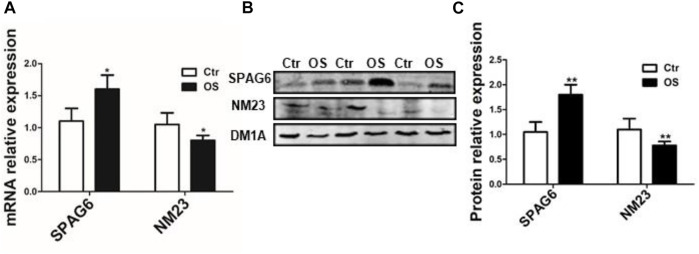
qRT-PCR and Western blot analysis of SPAG6 and NM23 in osteosarcoma and adjacent normal muscle tissues (control). **(A)** SPAG6 and NM23 mRNAs were detected by qRT-PCR. **(B)** SPAG6 and NM23 proteins were detected by Western blotting in osteosarcoma and adjacent normal tissues. **(C)** Quantitative analysis was conducted, and the results were normalized against the levels of α-tubulin (DM1A). The data were presented with ±s (*n* = 6). OS: osteosarcoma; Ctr: control (adjacent normal muscle tissues). *p < 0.05 *vs.* control group.

The positive expression rate of SPAG6 protein was 71.43% (30/42) in 42 cases of osteosarcoma tissues and 33.33% (4/12) in 12 cases of osteochondroma tissues, with *p* < 0.05 for all. The staining of SPAG6 was located in the cytoplasm and nucleus of osteosarcoma and control tissues (Figure 1A, B). The positive expression rate of NM23 protein was 35.71% (15/42) in 42 cases of osteosarcoma tissues and 58.33% (7/12) in 12 cases of osteochondroma tissues, with *p* < 0.05 for all. The staining of NM23 was located in the cytoplasm of osteosarcoma and control tissues (Figures 1C, D). To further validate our results, the mRNA and protein expression levels of SPAG6 and NM23 were detected by RT-PCR and Western blot analyses in six cases of osteosarcoma and adjacent normal tissues. We found that mRNA and protein levels of SPAG6 were significantly higher than those of the adjacent normal tissues (Figure 2A), while the expression of NM23 was lower in osteosarcoma tissues than in controls (Figure 2A). As shown in Figures 2B,C, the results of the Western blot analysis indicated that the relative intensity of SPAG6 and NM23 protein blots was 1.80- and 0.78-fold greater in osteosarcoma tissues than in control tissues, with *p* < 0.05 for all.

### The association between aberrant expression of SPAG6 and NM23 and clinicopathological features in human osteosarcoma patients

Among the 42 patients with human osteosarcoma, 25 were males and 17 were females; the age ranged from 6 to 75 years, and the average age was 19.35 years. A total of 22 patients were younger and 20 patients were older than 25 years. There were 19 lesions in adjacent knees, 8 in the humerus, 7 in jaws, and 8 in other bones. According to multiple distinct histological subtypes, human osteosarcoma is classified as conventional (osteoblastic), chondroblastic, fibroblastic, telangiectatic, small cell, surface, and secondary subtypes (Assi et al., 2021). In this study, there were osteoblastic subtype (*n* = 19), fibroblastic subtype (*n* = 10), chondroblastic subtype (*n* = 10), and others subtypes (*n* = 3). The diameter of the lesions was longer than 5.0 cm in 25 cases and shorter than 5.0 cm in 17 cases. About metastasis, especially to lungs, 31 cases were found metastasized. A total of six cases were low-potential malignancy (G1) and 36 cases were high-potential malignancy (G2). With regard to Enneking surgery stages, 8 cases were stage Ⅰ, 14 cases were stage Ⅱ, and 20 cases were stage Ⅲ. As for the 12 osteochondroma patients, 7 were males and 5 were females, and the age ranged from 8 to 42 years with a mean age of 18.5 years.

To further study the clinical significance of the aberrant expression of SPAG6 and NM23 proteins, the relationship between the expression of SPAG6 and NM23 and clinicopathological features in human osteosarcoma patients was analyzed. The expression of SPAG6 protein was positive while that of NM23 was negative, associated with distant metastasis, pathological grade, and Enneking stage of osteosarcoma patients, with *p* < 0.05 for all. But the expression of SPAG6 and NM23 proteins was not associated with patient’s gender, age, tumor location, histological types, and diameter (*p* > 0.05) (Table 2).

**TABLE 2:** Expression of SPAG6 in OS and the relationship with clinicopathological parameters (n = 42).

Parameter	Case	SPAG6		*p*-value	NM23		*p*-value
		+	-		+	-	
Gender							
Male	25	19	6	0.43	8	17	0.54
Female	17	11	6		7	10	
Age (year)							
<19	22	16	6	0.85	8	14	0.93
≥19	20	14	6		7	13	
Location							
Knee	19	13	6	0.74	7	12	0.67
Humerus	8	6	2		2	6	
Jaw	7	5	2		2	5	
Others	8	6	2		4	4	
Histological type							
Osteoblast	19	14	5	0.81	6	13	0.18
Fibroblast	10	8	2		2	8	
Chondroblast	10	6	4		6	5	
Others	3	2	1		2	1	
Diameter (cm)							
<5.0	25	19	6	0.49	8	17	0.54
≥5.0	17	11	6		7	10	
Distant metastasis							
Yes	31	27	4	<0.01	8	23	0.02
No	11	3	8		7	4	
Pathological grade							
G1	6	2	4	0.03	5	1	<0.01
G2	36	28	8		10	26	
Enneking stage							
I	8	2	6	<0.01	6	2	<0.01
II	14	10	4		6	8	
III	20	18	2		3	17	

### Prognosis and multivariate analysis

**FIGURE 3:**
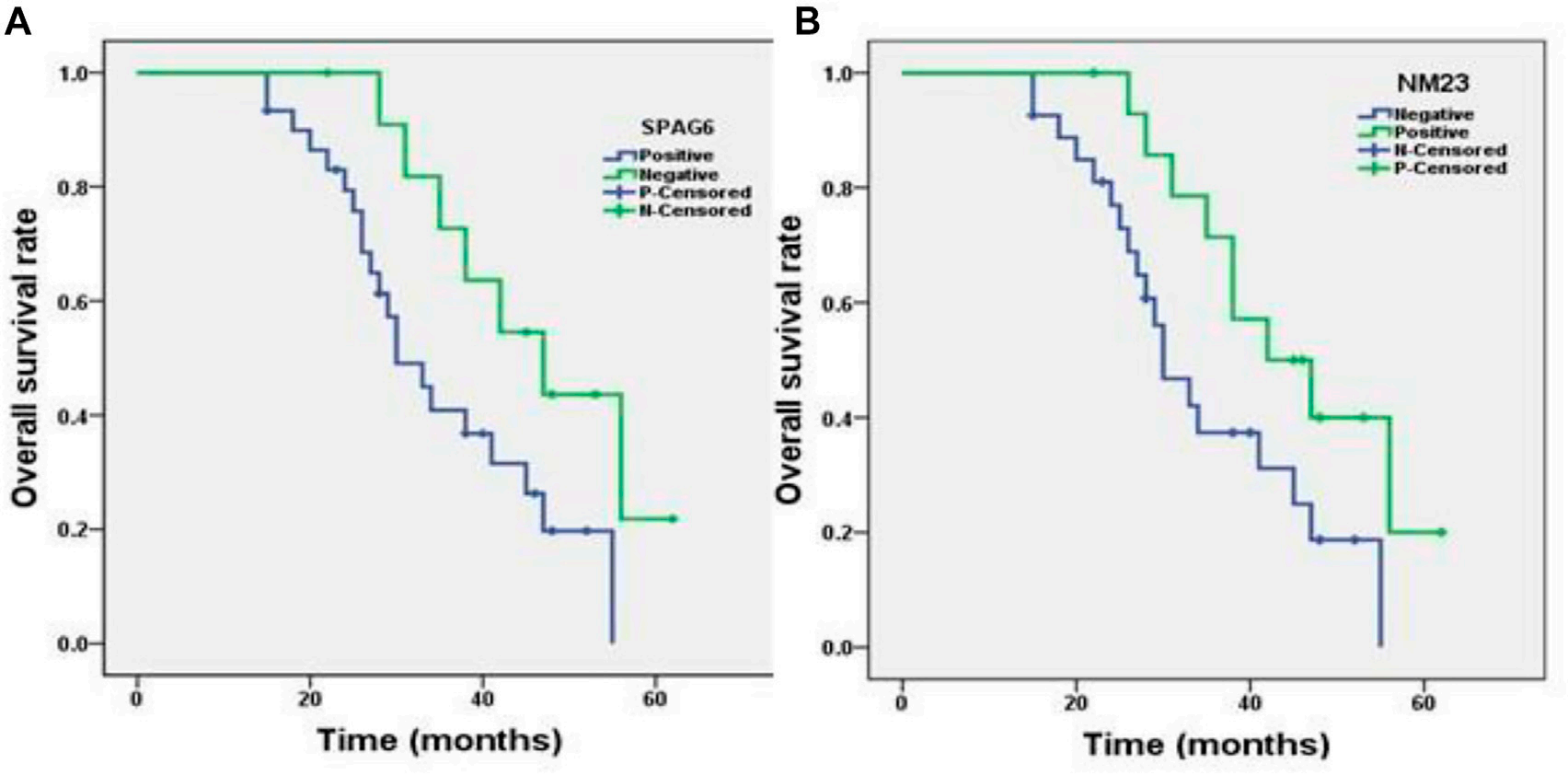
Kaplan−Meier survival analysis of patients with osteosarcoma (*n* = 42). **(A)** Correlation of the SPAG6 protein expression level with the overall survival time of patients with osteosarcoma. The y-axis represents the percentage of patients, the x-axis represents their survival in months, and the green line represents SPAG6^+^ patients with a worse survival trend than the blue line representing SPAG6^-^ (*p* < 0.05). **(B)** Correlation of the NM23 protein expression level with the overall survival time of patients with osteosarcoma, and the green line represents NM23^+^ patients with a better survival trend than the blue line representing NM23^-^ patients (*p* < 0.05).

Our results showed that the overall mean survival time of osteosarcoma patients with positive expression of SPAG6 (35.08 ± 2.58 months) was significantly shorter than that with negative expression of SPAG6 (46.69 ± 2.35 months), while the overall mean survival time (45.30 ± 3.47 months) of the NM23 positive expression group was longer than that of the NM23 negative expression group (34.56 ± 2.77 months), with *p* < 0.05 for all. The Kaplan−Meier curve analysis (log-rank test) results showed that the overall survival rate of osteosarcoma patients with SPAG6 positive expression was significantly less than that with SPAG6 negative expression (Figure 3A), while the rate was adverse in NM23 expression (Figure 3B) with *p* < 0.05 for all.

The expression of SPAG6 and NM23, distant metastasis, pathological grade, and Enneking stages were found to be independent prognostic factors of osteosarcoma using the Cox multivariate analysis method (*p* < 0.05 for all, Table 3).

**TABLE 3:** Multivariate survival analysis of 42 patients with osteosarcoma.

Covariate	B	SE	Sig	HR	HR 95%CI
Pathological degree	−39.695	18.928	0.036	0.000	0.000–0.075
Distant metastasis	−42.559	18.993	0.025	0.000	0.000–0.005
Enneking stage	0.897	0.395	0.023	1.131	1.131–5.320
SPAG6	1.971	0.873	0.024	1.298	1.298–39.727
NM23	2.502	1.175	0.033	1.221	1.221–122.076

### Correlation of SPAG6 and NM23 in human osteosarcoma

In thirty cases of the SPAG6 positive expression group, there were only six cases of NM23 positive expression in human osteosarcoma tissues. But in the twelve cases of the SPAG6 negative expression group, there were nine cases of NM23 positive expression in human osteosarcoma tissues. The negative correlation between SPAG6 and NM23 proteins was found (r = 0.519 and *p* < 0.01).

## Discussion

Osteosarcoma displays a bimodal distribution, with the first peak at the age of 10–19 years and the second peak at 70–79 years (Savage and Mirabello, 2011). In teenage, it is often distributed in the metaphysis of the long bones nearby the knee joints, and the second region is the vicinity of the shoulder joints (distal femur > proximal tibia > proximal humerus) (Damron et al., 2007; Sasaki et al., 2019). In the elderly, it is often distributed in the axial skeleton and skull (Rickel et al., 2017). According to pathological subtypes, osteosarcoma is classified as osteoblastic, and the most popular subtypes are chondroblastic, fibroblastic, telangiectatic, small cell, surface, and secondary subtypes (Assi et al., 2021). Relapsing or metastatic patients with osteosarcoma have a dismal prognosis with a median overall survival of less than 8 months (Lagmay et al., 2016). NM23 plays a suppressing role in tumor metastasis (Carotenuto et al., 2015; Kim and Lee, 2021). It inhibits bone-specific metastasis by upregulating miR-660-5p in lung cancer and represses metastasis *via* redox regulation in breast cancer (Ai et al., 2020; Kim and Lee, 2021). In this study, we found NM23 was downregulated more in osteosarcoma tissues than the controls in mRNA and protein levels. Furthermore, it was negatively related to the pathological grade, distant metastasis, Enneking stage, and worst prognosis of patients with osteosarcoma.

SPAG6 was first inspected in human testicular tissue and is mainly expressed in the sperm, lungs, central nervous system, and inner ear (Hu et al., 2016; Liu et al., 2019). Functionally, SPAG6 is predominately involved in sperm maturation and nervous system development under normal physiological conditions in mammals (Li et al., 2017; Liu et al., 2019). Accumulating evidence has demonstrated that SPAG6 was identified as a novel cancer-testis antigen (Silina et al., 2011). Aberrant expression of SPAG6 in hematological malignancies, Burkitt lymphoma, and breast and non-small cell lung cancers may serve an important role in the occurrence and development of different human cancers by regulating the growth, apoptosis, invasion, and metastasis of tumor cells *via* AKT/FOXO, PTEN/PI3K/AKT, and other pathways (Jiang et al., 2019; Mijnes et al., 2019; Zheng et al., 2019; Zhang et al., 2020). So far, there are no reports about the clinical significance and correlation between SPAG6 expression and NM23 in osteosarcoma.

In the present study, we reported that SPAG6 was upregulated more in osteosarcoma tissues than the controls in mRNA and protein levels. Furthermore, research showed that the expression of SPAG6 was positively related to the pathological grade, distant metastasis, Enneking stage, and worst prognosis of patients with osteosarcoma. Also, the positive expression of SPAG6 indicated a shorter mean overall survival time (35.08 months) than SPAG6 negative expression (45.30 months). For children, where recurrent/refractory osteosarcoma was uniformly poor, there have been few new chemotherapy drugs, small-molecule targeted medicine, or immunotherapeutic agents found to treat osteosarcoma (Meyers et al., 2005; Lagmay et al., 2016). Our research also showed that SPAG6 and NM23 were positively correlated with each other. We deduced that SPAG6 may promote distant metastasis by suppressing NM23 and lead to worse prognosis in osteosarcoma. Some studies found that SPAG6 is also a promising anti-cancer therapeutic candidate (Li et al., 2017; Yin et al., 2018).

Our results suggest that SPAG6 may provide a potential tumor marker and a promising antitumor therapeutic target. To some extent, combined detection of SPAG6 and NM23 can indicate the biological behavior of osteosarcoma cells, thus giving a selection of targeted therapies. However, our study only detected mRNA and proteins in human osteosarcoma tissues; the molecular mechanism of SPAG6 regulating NM23 will be studied in our future research.

## Data Availability

The original contributions presented in the study are included in the article/Supplementary Material; further inquiries can be directed to the corresponding authors.
