# Supplementary material for: Aberrant expression of SPAG6 and NM23 predicts poor prognosis of human osteosarcoma
Source: Front Genet. 2022 Sep 19;13:1012548. doi: 10.3389/fgene.2022.1012548 (PMC9527292; doi:10.3389/fgene.2022.1012548)
Supplement: Supplementary file 1 [file Presentation1.PPTX]

## Slide 1
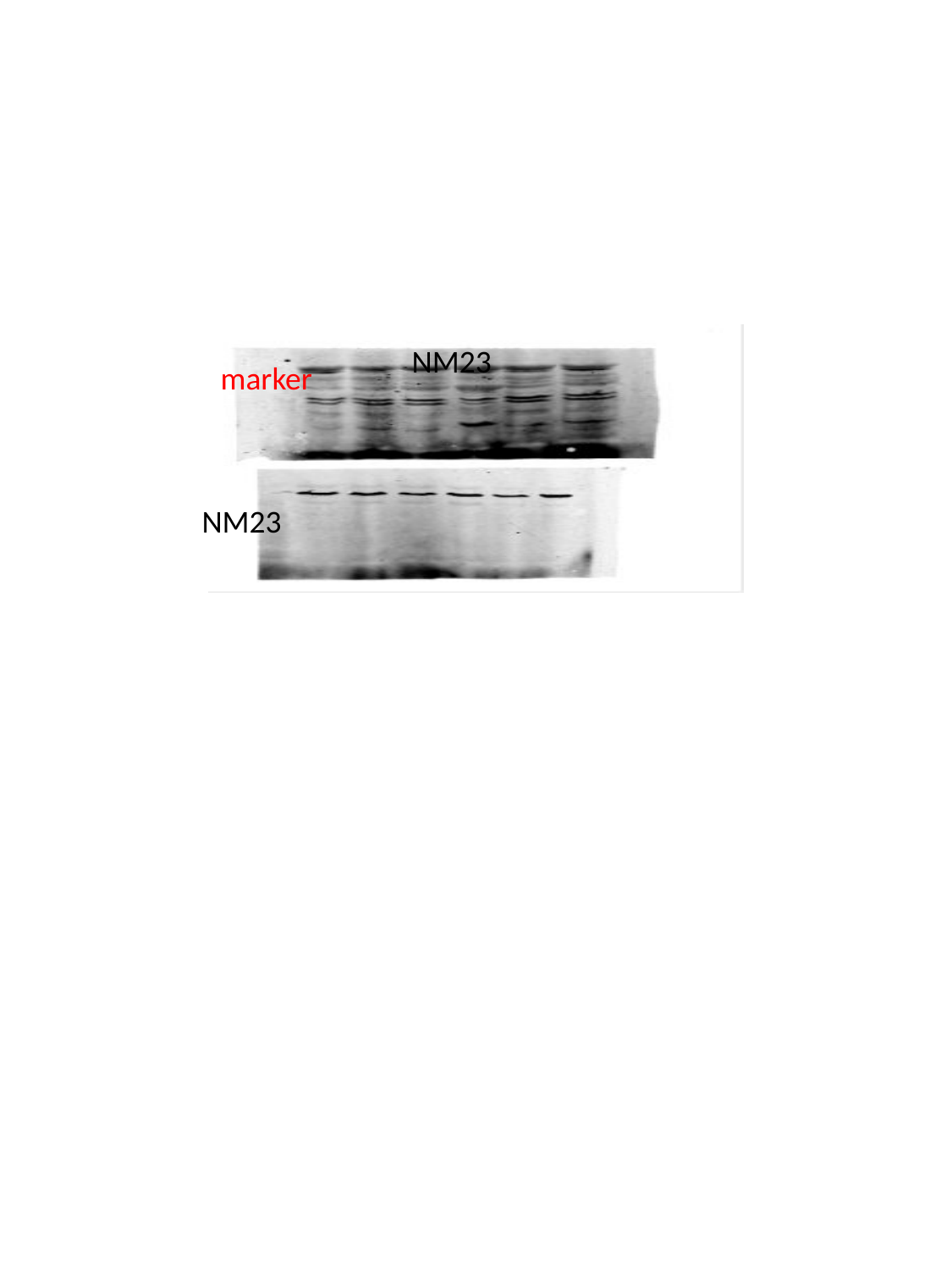

NM23
marker
NM23

## Slide 2
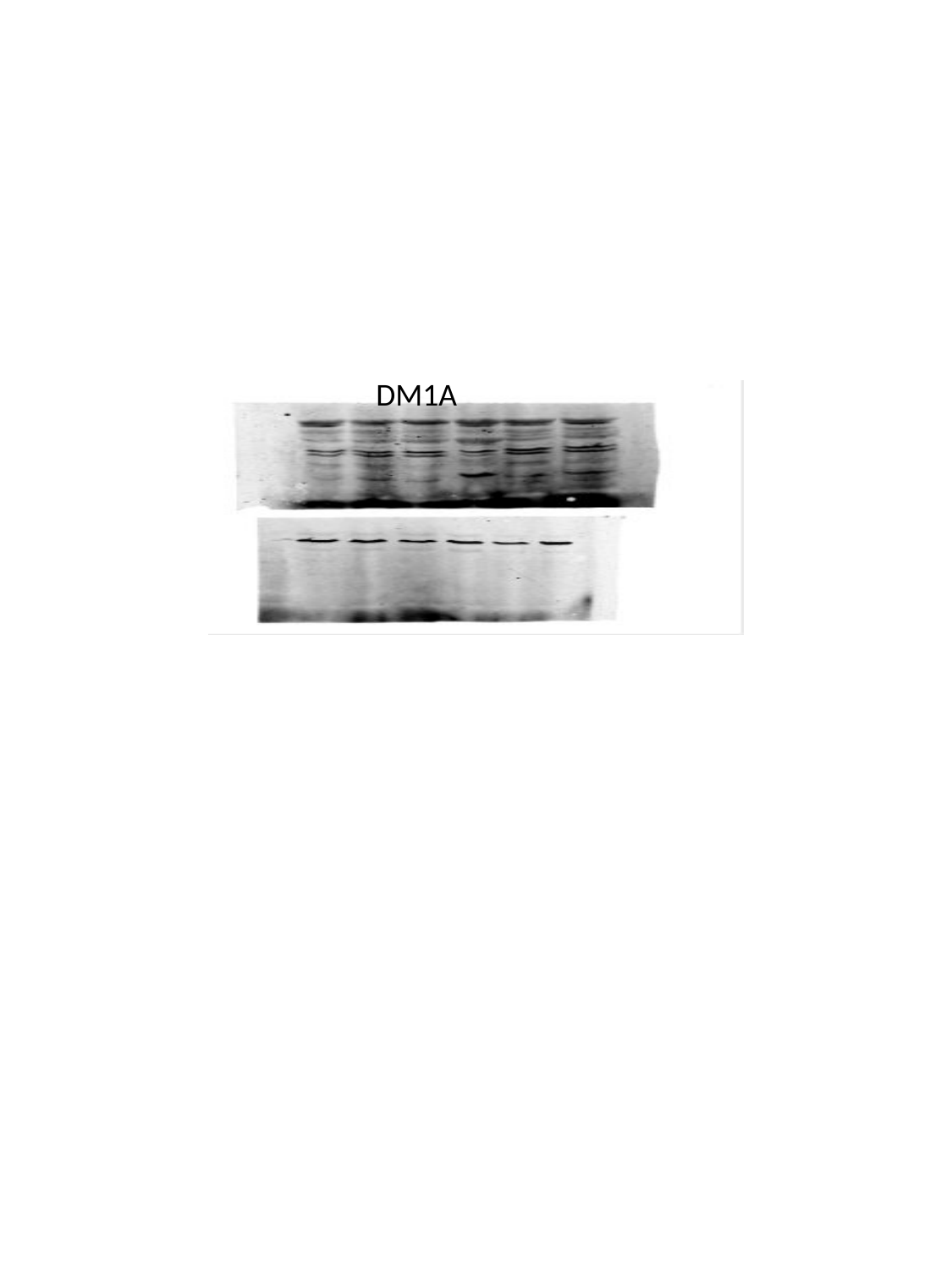

DM1A

## Slide 3
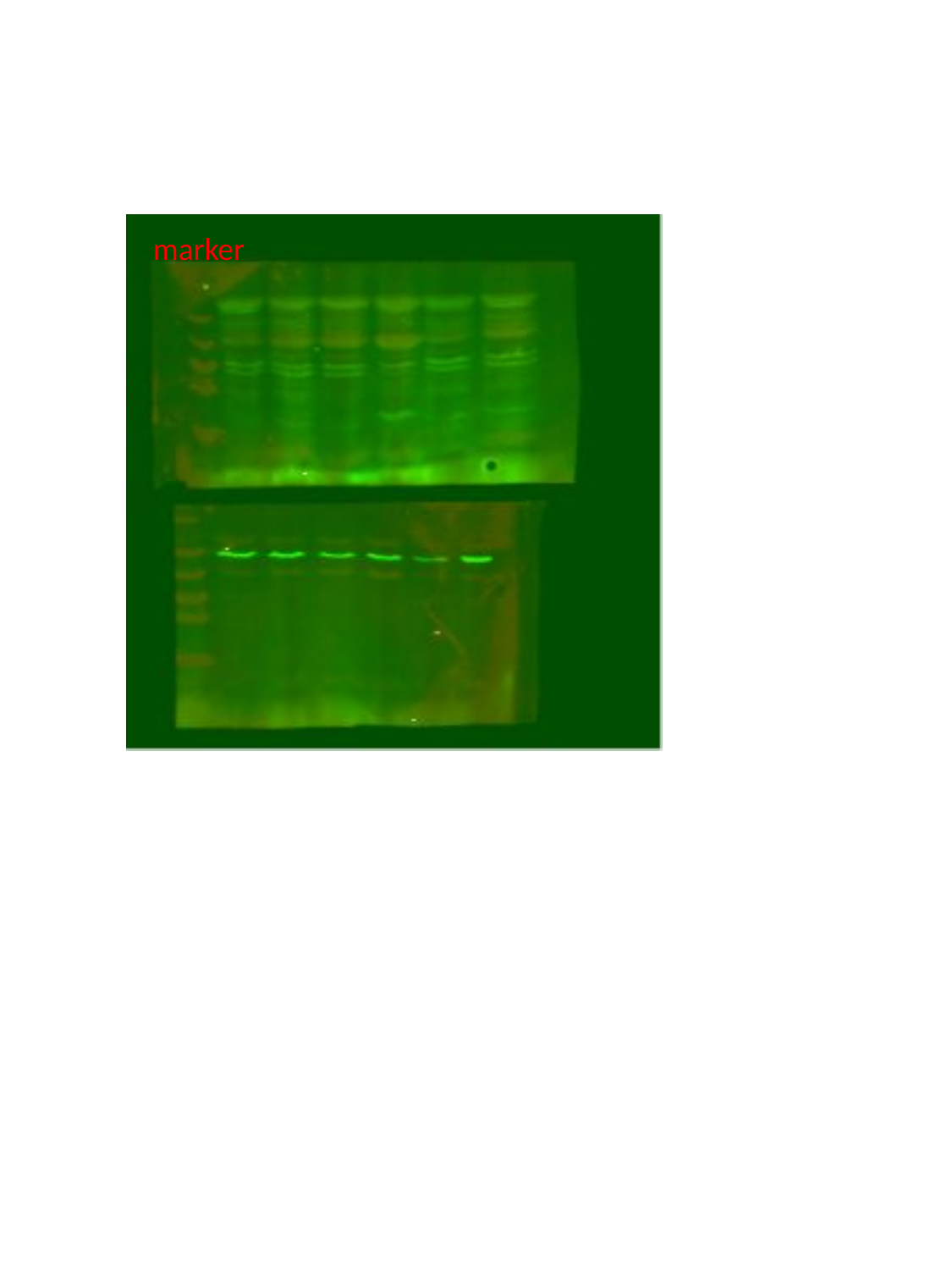

marker

## Slide 4
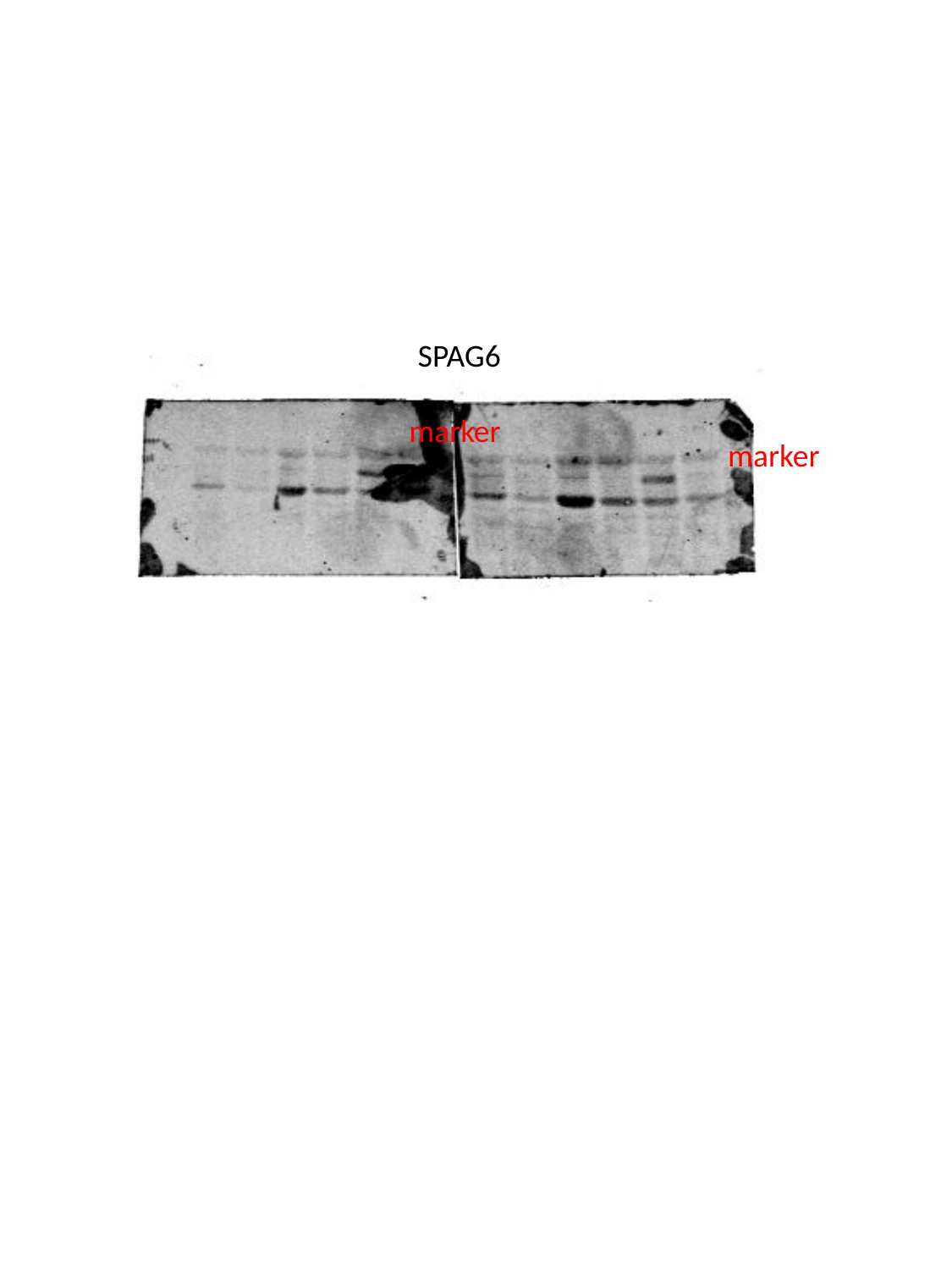

SPAG6
marker
marker

## Slide 5
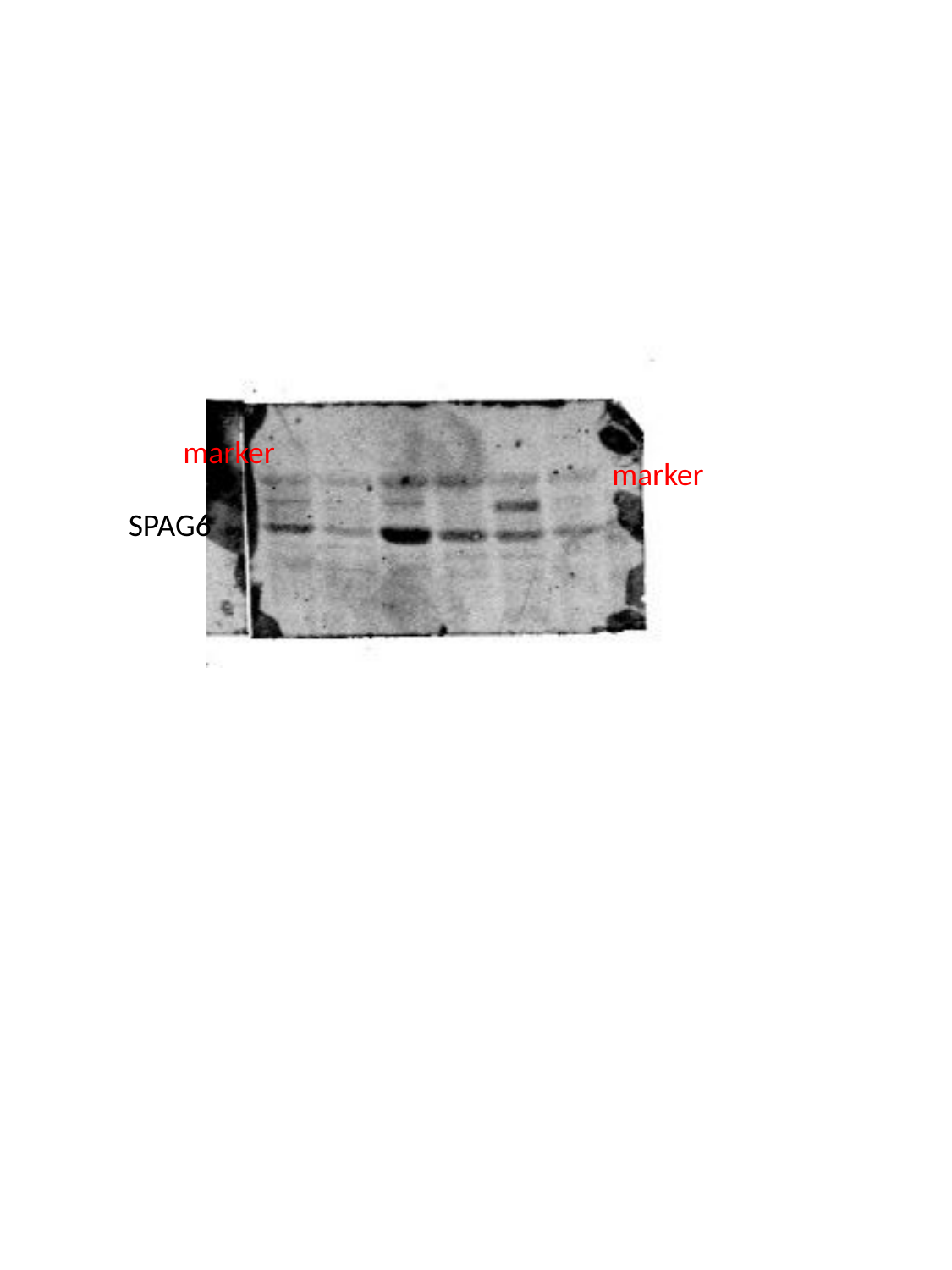

marker
marker
SPAG6

## Slide 6
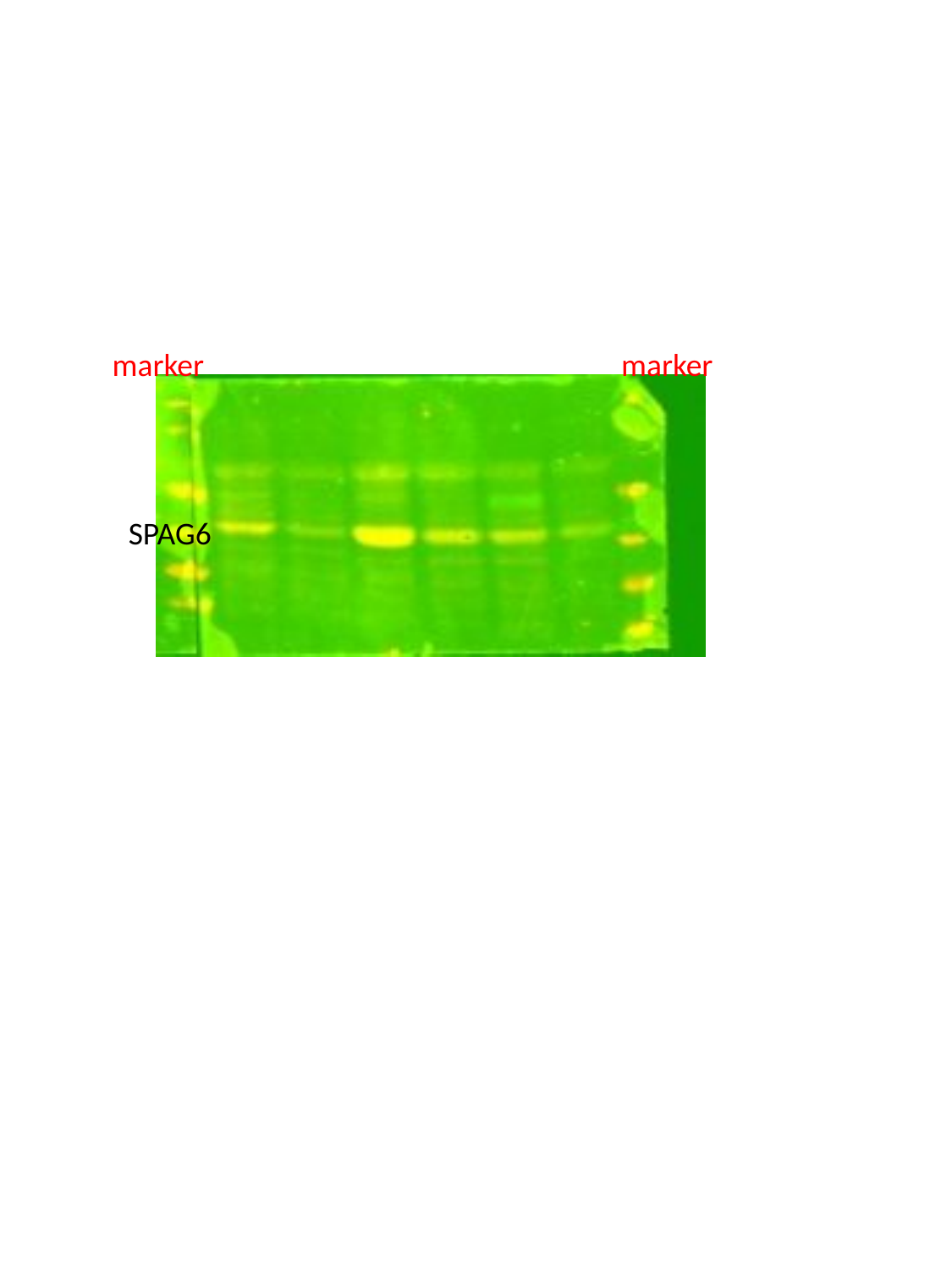

marker
marker
SPAG6
